# Charge density and particle size effects on oligonucleotide and plasmid DNA binding to nanosized hydrotalcite

**DOI:** 10.1186/1559-4106-8-8

**Published:** 2013-03-21

**Authors:** Brian A Sanderson, Drew S Sowersby, Sergio Crosby, Marcus Goss, L Kevin Lewis, Gary W Beall

**Affiliations:** 1 Department of Chemistry and BiochemistryTexas State University78666San MarcosTXUSA; 2 Physics Department, Faculty of ScienceKing Abdulaziz University21589JeddahSaudi Arabia

**Keywords:** Hydrotalcite, DNA, Gene therapy, Molecular modeling, Charge density

## Abstract

Hydrotalcite (HT) and other layered double metal hydroxides are of great interest as gene delivery and timed release drug delivery systems and as enteric vehicles for biologically active molecules that are sensitive to gastric fluids. HT is a naturally occurring double metal hydroxide that can be synthesized as a nanomaterial consisting of a brucite structure with isomorphous substitution of aluminum ions. These positively charged nanoparticles exhibit plate-like morphology with very high aspect ratios. Biomolecules such as nucleic acids and proteins form strong associations with HT because they can associate with the positively charged layers. The binding of nucleic acids with HT and other nanomaterials is currently being investigated for potential use in gene therapy; however, the binding of specific nucleic acid forms, such as single- and double-stranded DNA, has been little explored. In addition, the effects of charge density and particle size on DNA adsorption has not been studied. In this paper, the binding of different forms of DNA to a series of HTs prepared at different temperatures and with different anion exchange capacities has been investigated. Experiments demonstrated that HTs synthesized at higher temperatures associate with both single- and double-stranded oligomers and circular plasmid DNA more tightly than HTs synthesized at room temperature, likely due to the hydrothermal conditions promoting larger particle sizes. HT with an anion exchange capacity of 300 meq/100 g demonstrated the highest binding of DNA, likely due to the closer match of charge densities between the HT and DNA. The details of the interaction of various forms of DNA with HT as a function of charge density, particle size, and concentration are discussed.

## Background

Hydrotalcite (HT) is a double metal hydroxide clay particle that is abundant in nature and is readily synthesized in the laboratory [1, 2]. HT has recently gained much attention because of its many and varied applications, such as support for catalysts, anion exchangers [3], water treatment [4], flame retardants, sorbents and separation of proteins and enzymes [5–7], time release pharmaceutical [8, 9], enteric delivery systems [10], and cosmetic uses [11–13]. The general formula for HT is expressed as [M^II^_1-x_M^III^_x_(OH)_2_]A^n-∗^H_2_O, where M^II^ is a divalent metal cation (Mg^2+^), M^III^ is a trivalent metal cation (Al^3+^), and A^n-^ is the interlayer anion (Cl^-^) [4]. HT layers gain a positive charge by isomorphous substitution of Al^3+^ for Mg^2+^, which is compensated by interlayer anions and water [1, 11, 14]. These interlayer anions, especially halides and nitrate, can be exchanged with anions in external solution, including biomolecules, for use as a drug and gene delivery system [11, 14]. The HT-biomolecule nanohybrids can then be completely decomposed by acidic body fluid once they reach the delivery point, releasing the adsorbed drug or gene [13]. In nature and in laboratory prepared samples, if precautions are not taken to isolate the synthesis from contact with air, the most common anion is carbonate. The carbonate ion binds the layers together so strongly that exchange with biomolecules is almost impossible. Unfortunately all commercially available HT’s are sold in the carbonate form and have high charge density.

HT can by synthesized at different temperatures and different anion exchange capacities (AEC) from e.g., 100–500 meq (milliequivalents or milimoles of charge/100 g), giving rise to various particle sizes and electrostatic forces between the layers and anions [13]. Direct synthesis is the most common method of producing HT, which involves the precipitation of HT in an aqueous solution of metal salts (MgCl_2_ and AlCl_3_), water, and sodium hydroxide (NaOH), bringing the pH to 10 [14]. When excess divalent metal is added, HT precipitates at a lower pH, which limits the uptake of CO_2_ by the reaction mixture. The initial HT forms an infinite two-dimensional layer due to the positively charged octahedrally coordinated metal ions sharing edges [1, 14]. Electrostatic forces between layers and interlayer anions then help form the three-dimensional structure of HT [14]. Interlayer spacing (d-spacing) between layers varies depending on the Mg^2+^/Al^3+^ mole ratio, exchangeable anion, level of hydration, and size/geometric structure of the intercalated anions [12, 14].

Interactions between HT and biomolecules, such as cytidine-, adenosine-, and guanosine monophosphate, as well as RNA and DNA, have been extensively investigated [2, 13]. HT and DNA interactions are particularly important for the application of gene therapy since HT can be used as a non-toxic vector to transfer genes through cell membranes into cells and organs [1, 13]. Once the HT-DNA is in the organ of choice, the DNA can be released from the interlayer space with a change of pH, e.g., after adding carbonate ions [13, 14]. Most HT-DNA interactions have been investigated using short single- and double-stranded DNA (ssDNA and dsDNA) fragments. Interactions between circular double-stranded plasmid DNA (pDNA), the major form of DNA used in gene therapy, and HT has been little explored [14].

HT-DNA interactions can be analyzed quantitatively by using DNA’s strong absorbance of ultraviolet (UV) light at 260 nm to determine the concentration of DNA in solution [15]. DNA (single-stranded, double-stranded, or plasmids) can be mixed with HT (varying concentrations), vigorously shaken for sufficient time to allow the equilibrium binding of DNA onto the HT, and then centrifuged to pellet the bound DNA and HT [15–17]. The supernatant can then be analyzed for residual absorbance at 260 nm to calculate the concentration of unbound DNA [15]. Reconstruction and ion exchange methods can also be used to examine the interactions between HT and DNA [2].

In this study, DNA (single-stranded, double-stranded, and plasmid) was bound to a series of different HT samples to determine the effects of charge density, particle size, concentration, and competing anions on adsorption. The results demonstrate that binding of DNA is critically dependent on both the size and charge density of the clay platelets. The data further indicate that, althought HTs are frequently prepared at RT, this temperature is not optimum. Higher synthesis temperatures produced larger particles with improved DNA binding capacities.

## Methods

### Hydrotalcite and DNA

Hydrotalcite was prepared through the precipitation of MgCl_2_, H_2_O, and AlCl_3_ at different ratios to obtain the desired AEC (100–500 meq./100 g). MEQ is milliequivalents or millimoles of charge (e.g., Mg^2+^ would have 2000 meq/mole). NaOH was subsequently added to bring the pH to 10. The whole mixture was then hydrothermally heated in a Parr reactor at temperatures of 80, 130, or 150°C. The oligonucleotides Pvu4a (sequence: AAATGAGTCACCCAGATCTAAATAA) and its complement, cPvu4a (sequence: TTATTTAGATCTGGGTGACTCATTT), were purchased from BioServe Biotechnologies, Ltd. The 50 bp ladder and Quick-Load 1 kb DNA ladders were purchased from New England BioLabs. Agarose powder was purchased from EMD Chemicals and ethidium bromide was obtained from IBI-Shelton Scientific.

### Equipment

The HT was characterized by x-ray diffraction utilizing a Bruker D-8 diffractometer employing a Cu K α x-ray source. Centrifugation was performed using an Eppendorf microcentrifuge 5415 D. Scanning electron microscopy was conducted utilizing a FEI Helios Nanolab 400 at 10Kv. A BioRad SmartSpec 3000 spectrophotometer and Hoefer DyNA Quant 200 Fluorometer were used for spectroscopic measurements. Gel electrophoresis was performed using a Life Technologies Horizon 11*14 electrophoresis apparatus and a Thermo Electron Corporation EC105 power supply.

### Sample preparation

#### Preparation of double-stranded oligonucleotide DNA

The double-stranded oligonucleotide DNA was prepared by mixing Pvu4a, cPvu4a, and double-deionized water in a final volume of 2.2 ml and concentration of 1,500 ng/μl. The solution was placed in a heating block for 5 min at 100°C. Annealing then occurred at RT for 30 min. The ss- and dsDNA samples were then analyzed using agarose gel electrophoresis with 4% agarose and 1X TBE (Tris, borate, EDTA) gel running buffer for 1 h at 200 volts. The gel was visualized using ethidium bromide and photographed with a Kodak Digital Science Image Station 440CF system. The molecular weight standards employed were 5 bp ladder and 50 bp ladder (New England Biolabs).

#### Preparation of plasmid DNA

A pRS316 maxiprep was performed as described by the Machesky protocol [18], except for the following changes: *Escherichia coli* LKL37a strains containing the pRS316 plasmid were initially transferred from LB plates containing 100 αg/ml ampicillin (Amp) into 500 ml of LB + Amp broth and shaken for 24 h at 37°C. The solution was then centrifuged at 7000 rpm for 5 min and the cell pellet was resuspended in 10 ml of solution I. Once the cell suspension was homogeneous, 10 ml of solution II was aliquoted to the suspension. Then 7.5 ml of solution III was added followed by centrifugation at 14,000 rpm. The supernatant was removed, mixed with 20 ml of isopropanol, and centrifuged at 14,000 rpm for 5 min. The pellet was washed with 70% ethanol and then dried for 30–60 min. The pellet was dissolved in 1.2 ml of TE and 5 μl of RNase A (20 mg/ml) was added, followed by incubation at 37°C for 30 min. The resulting DNA was cleaned by precipitation with isopropanol and sodium acetate and washing with 70% ethanol. The concentration of plasmid DNA was then determined using fluorescence spectroscopy and analysis by gel electrophoresis as described above on a 0.9% agarose gel run at 150 V for 1 h.

#### Preparation of DNA-hydrotalcite

HT-DNA solutions were prepared by mixing HT at concentrations of 0.08, 0.4, 2, or 10 mg/ml, DNA (33 ng/μl for ssDNA or 50 ng/μl for dsDNA), and double-deionized water in a final volume of 300 μl. The mixture was gently shaken (900 rpm) for 5 min and then centrifuged at 16,100 × g for 5 min at RT.

### Analysis of DNA-hydrotalcite Adsorption

The upper 60 μl of each 300 μl sample was used to measure the absorbance of unbound DNA using UV light spectroscopy at 260 nm (A_260_) and the average of each HT series was calculated. The percent DNA adsorbed on the HT was calculated by subtracting the average absorbance of unbound DNA for each sample from the original DNA-only absorbance and dividing the difference by the DNA-only absorbance. 1.5 ml Eppendorf Flex-tubes were used for all assays since these tubes were previously shown to leach fewer UV light-absorbing chemicals than other brands [19]. Microsoft Excel software was utilized to graphically analyze the adsorption results between each HT series.

### X-ray diffraction

The x-ray analysis was carried out on a Bruker D-8 diffractometer utilizing Cu Kα radiation. The patterns were scanned from 2 to 60 degrees 2 theta at a step size of 0.03 degrees and step time of 2 seconds.

### Scanning electron microscopy

The samples were investigated using the field emission scanning electron microscopy (FESEM), Helios Nano lab 400 equipped with energy dispersive x-ray (EDX) spectrometer. The maximum accelerating voltage was 30 KV while the working distance was 4 mm.

## Results and discussion

### Characterization of the hydrotalcites

The HT’s were prepared at various levels of isomorphous substitutions of Al for Mg. The following are formulas for the targeted levels of charge density (Table  1).

**Table 1:** Theoretical and actual synthesized compositions of hydrotalcites used in this study

Target meq.	General formula for hydrotalcite	Actual formula	Actual meq.
100 meq/100 g	[M^II^_1-x_M^III^_x_ * (OH)_2_]A^n-^	[Mg^2+^_.94_Al^3+^_.06_(OH)_2_]Cl^-^_.06_	103
200 meq/100 g	[M^II^_1-x_M^III^_x_ * (OH)_2_]A^n-^	[Mg^2+^_.88_Al^3+^_.12_(OH)_2_]Cl^-^_.12_	205
300 meq/100 g	[M^II^_1-x_M^III^_x_ * (OH)_2_]A^n-^	[Mg^2+^_.82_Al^3+^_.18_(OH)_2_]Cl^-^_.18_	308
400 meq/100 g	[M^II^_1-x_M^III^_x_ * (OH)_2_]A^n-^	[Mg^2+^_.77_Al^3+^_.23_(OH)_2_]Cl^-^_.23_	393
500 meq/100 g	[M^II^_1-x_M^III^_x_ * (OH)_2_]A^n-^	[Mg^2+^_.71_Al^3+^_.29_(OH)_2_]Cl^-^_.29_	496

**Figure 1:**
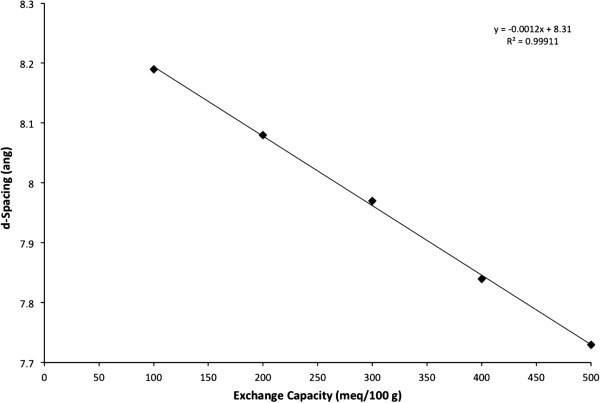
**Plot of x-ray d-spacing for a series of hydrotalcites of different exchange capacities grown at 150°C.**

The samples were filtered, dried and characterized by x-ray diffraction. The main parameter that changes with charge density in HT’s is the spacing between the plates. This can be probed by measuring the d-spacing of the 00 l x-ray reflection. The trend of d-spacing against exchange capacity for HT’s grown at 150°C is given in Figure  1. The d-spacings were 8.19, 8.08, 7.97, 7.84, and 7.73 angstroms for 100, 200, 300, 400, and 500 meq/100 g, respectively. It can be seen that there is a linear relationship between d-spacing and level of aluminum substitution and that the higher charge binds the layers together more tightly.

**Figure 2:**
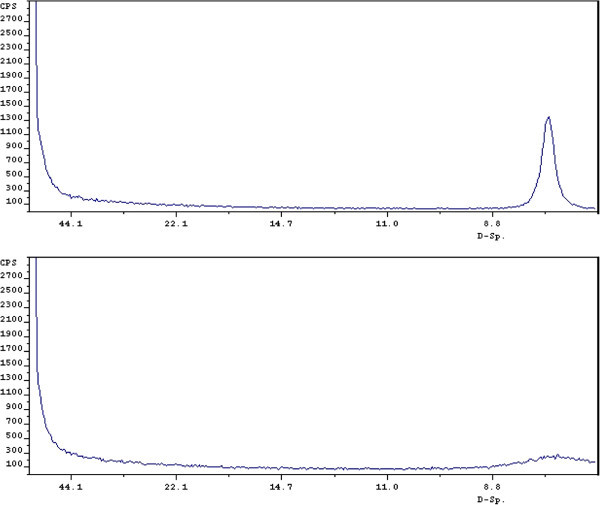
**Hydrotalcite 300 meq./100 g. grown at RT or 150°C.** Upper panel, 150°C; Lower panel, 25°C.

**Figure 3:**
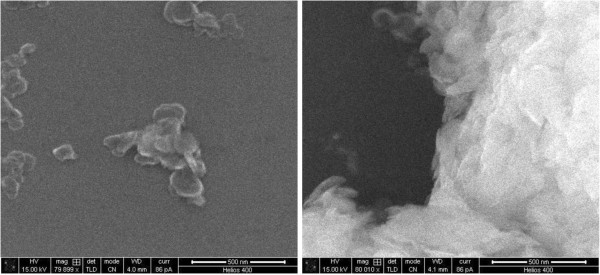
**Scanning Electron Microscope images of 400 meq/100 g HT grown at 25°C(left) and 150°C(right).**

The second point of interest is found in comparing the x-rays of material grown at RT and 150°C. Figure  2 contains these x-ray patterns and it can be seen that the basal peak in the RT material is much broader, indicating smaller particle size. The assumption that hydrothermal conditions are necessary to produce larger plates appears to be correct, but they may actually lead to particles that are too large to give clear suspensions. The Scherrer [20] equation has been utilized to calculate the average particle size of the RT and 150°C material and yields 100 and 390 nm, respectively. This would mean that the 150°C material would have a surface area 14 times that of the RT material. These numbers are in qualitative agreement with the scanning electron microscope images of HT grown at 25°C and 150°C depicted in Figure  3. In the 25°C material there are very few particles of appreciable size with large agglomerates of amorphous looking masses and occasional discrete particle in the 50 to 100 nanometer range, while in the 150°C material there are a large number of particles that range in size from 200 to 400 nm.

### Characterization of double-stranded oligonucleotide DNA

**Figure 4:**
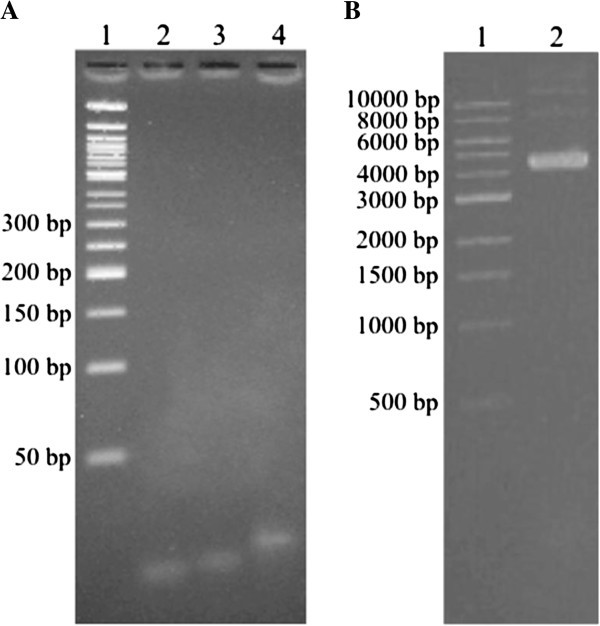
**(A) Analysis of single- and double-stranded oligonucleotide DNAs by agarose gel electrophoresis.***Lane 1,* 50 bp DNA ladder; *lane 2*, Pvu4a (400 ng); *lane 3*, cPvu4a (400 ng); *lane 4*, Pvu4a + cPvu4a after annealing. (**B**) pRS316 plasmid DNA preps are composed primarily of fast-migrating supercoiled DNA. *Lane 1*, 1 kb DNA ladder; *lane 2*, pRS316 DNA.

Two single-stranded 25 mer oligonucleotide DNAs, Pvu4a and cPvu4a, were employed for these studies. For the double-stranded DNA, Pvu4a and cPvu4a, which have complementary sequences, were annealed to each other. The double-stranded DNA was prepared by mixing both oligomers with equal mass in ddH_2_O, heating to 100°C, and incubating at RT. The dsDNA was then verified using 4% agarose gel electrophoresis in conjunction with ethidium bromide staining to analyze migration compared to each single-stranded oligomer (Figure  4A). Lanes 2 and 3, which contained Pvu4a and cPvu4a respectively, both showed broad bands at ~ 25 nt. The dsDNA can be seen in lane 4, where this larger species migrated slower than either single-stranded oligomer.

### Characterization of plasmid DNA

Plasmid maxipreps were performed using *E. coli* LKL37 a strains transformed with plasmid pRS316, which is a 4,887 bp cloning vector [21]. The composition of the plasmid DNA was assessed by analysis on a 0.9% agarose gel (Figure  4B). Lane 2 indicates that circular supercoiled DNA is the dominant species, which is typical for plasmid DNA isolated from bacterial cells [22].

### Measurement of DNA binding to HT

**Figure 5:**
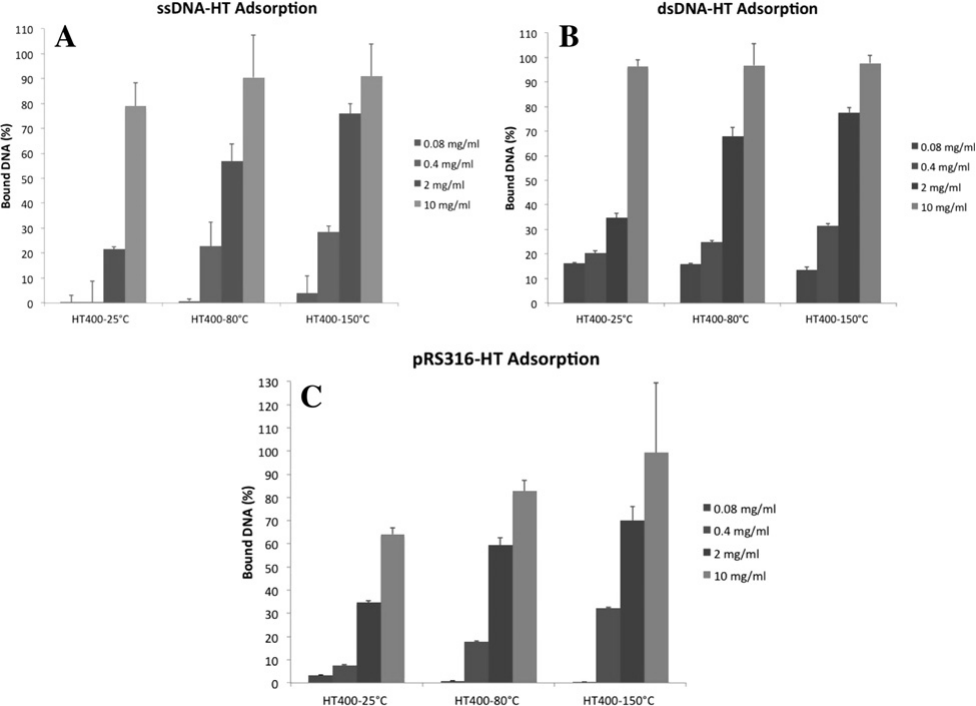
**Percent DNA bound to 0.08 mg/ml, 0.40 mg/ml, 2.0 mg/ml, and 10.0 mg/ml hydrotalcite.** Percent DNA bound = initial DNA absorbance minus absorbance of unbound DNA divided by initial DNA absorbance. Error bars indicate standard deviations. **A**, Single-stranded oligonucleotide DNA adsorption onto HT. **B**, Double-stranded oligonucleotide DNA adsorption onto HT. **C**, Plasmid, pRS316, adsorption onto HT.

Each mixture of DNA and HT was measured at 260 nm to determine the absorbance of unbound DNA in the supernatant after centrifugation. The percent DNA bound was then calculated by subtracting the final absorbance (representing unbound DNA) from the initial absorbance and then dividing by the initial DNA absorbance. The average percent DNA bound and standard deviations based on 5 trials were then graphed (Figure  5). In contrast to some nanomaterials such as montmorillonite clays, hydrotalcites do not absorb UV light at 260 nm [15]. Thus, all absorbance remaining in the supernatant must arise from unsedimented (unbound) DNA.

HT at a concentration of 10 mg/ml had the highest DNA adsorption for both oligomers and plasmid DNA at all three temperatures of HT synthesis, with no significant difference between adsorption of both oligomers. For 2 mg/ml HT, adsorption significantly increased as temperature of HT synthesis increased from 25 to 150°C for all three nucleic acid samples. This increase in adsorption with increased growth temperature would indicate that the larger the particle the better the adsorption, which correlates well with the SEM images. Another factor may be that the finely divided RT precipitate has a strong tendency to adsorb CO_2_ from the air. Thermogravimetric/Mass Spec analysis demonstrated that this material contains approximately 20% replacement of carbonate for chloride while HT grown at 80°C or above only has traces of carbonate (data not shown).

**Figure 6:**
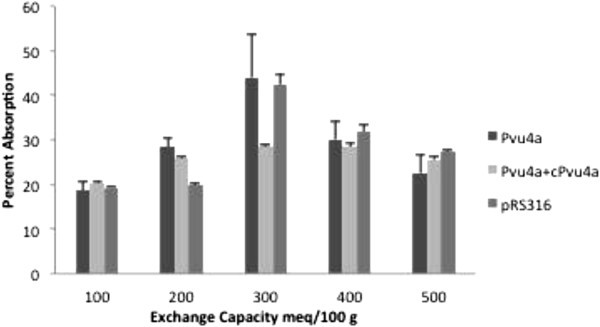
Percent adsorption of three types of DNA as a function of HT exchange capacity.

The adsorption of DNA onto HT at various AECs is shown in Table  2. There was no significant difference between the percentages of bound DNA when using 10 mg/ml HT, which indicates that the HT relative concentration was too high. All types of DNA yielded higher than 90% adsorption on to the HT. However, at 2 mg/ml HT the percent DNA bound for all three nucleic acids significantly increased from 100 to 300 meq/100 g at which point the sorption plateaued. At 0.40 mg/ml both the single-stranded oligomer and pRS316 had an increase in adsorption between 200 and 300 meq/100 g but then significantly decreased at 400 meq/100 g. The double-stranded oligomer with 0.40 mg/ml HT followed the same pattern as the results from 2 mg/ml HT, with an increase from 100 to 300 meq/100 g, then plateaued at 400 and showed a slight decrease at 500. Figure  6 gives a graphical representation of the adsorption of all types of DNA as a function of exchange capacity at 0.4 mg/ml HT. It is interesting to note that there is a definite maximum adsorption at 300 meq/100 g.

**Table 2:** **Adsorption of nucleic acids onto hydrotalcite with various anion exchange capacities**^**a**^

Bound DNA (%)					
[HT] (mg/ml)	100-150°C	200-150°C	300-150°C	400-150°C	500-150°C
Pvu4a					
0.08	5.3 ± 1.3%	8.3 ± 2.2%	19.2 ± 4.4%	6.6 ± 8.8%	12.1 ± 7.0%
0.4	18.7 ± 1.8%	28.4 ± 2.0%	44.0 ± 9.6%	30.1 ± 4.1%	22.6 ± 3.9%
2	41.0 ± 4.7%	59.1 ± 8.3%	78.2 ± 3.8%	78.3 ± 3.0%	76.5 ± 6.2%
10	92.7 ± 5.5%	94.2 ± 4.2%	95.5 ± 5.5%	92.9 ± 4.4%	96.2 ± 8.2%
Pvu4a + cPvu4a					
0.08	17.0 ± 0.5%	11.4 ± 0.2%	14.8 ± 0.2%	11.7 ± 0.1%	13.3 ± 0.4%
0.4	20.3 ± 0.4%	25.9 ± 0.4%	28.5 ± 0.5%	28.5 ± 0.6%	25.6 ± 0.6%
2	52.6 ± 2.5%	59.7 ± 4.7%	77.7 ± 7.0%	76.1 ± 3.7%	78.4 ± 6.6%
10	91.9 ± 6.0%	93.8 ± 4.0%	96.9 ± 4.6%	98.0 ± 4.7%	98.8 ± 3.5%
pRS316					
0.08	7.5 ± 0.1%	8.3 ± 0.1%	11.4 ± 0.4%	7.1 ± 0.1%	3.4 ± 0.1%
0.4	19.3 ± 0.3%	19.8 ± 0.4%	42.4 ± 2.2%	31.8 ± 1.6%	27.3 ± 0.4%
2	57.6 ± 1.8%	62.8 ± 3.3%	86.8 ± 6.3%	83.9 ± 6.5%	77.0 ± 7.7%
10	87.0 ± 5.3%	96.5 ± 9.9%	99.4 ± 30.4%	99.8 ± 17.6%	99.7 ± 22.1%

^a^ Nucleic acid samples include: Pvu4a, Pvu4a + cPvu4a, and pRS316. Numbers shown indicate averages ± standard deviations.

**Figure 7:**
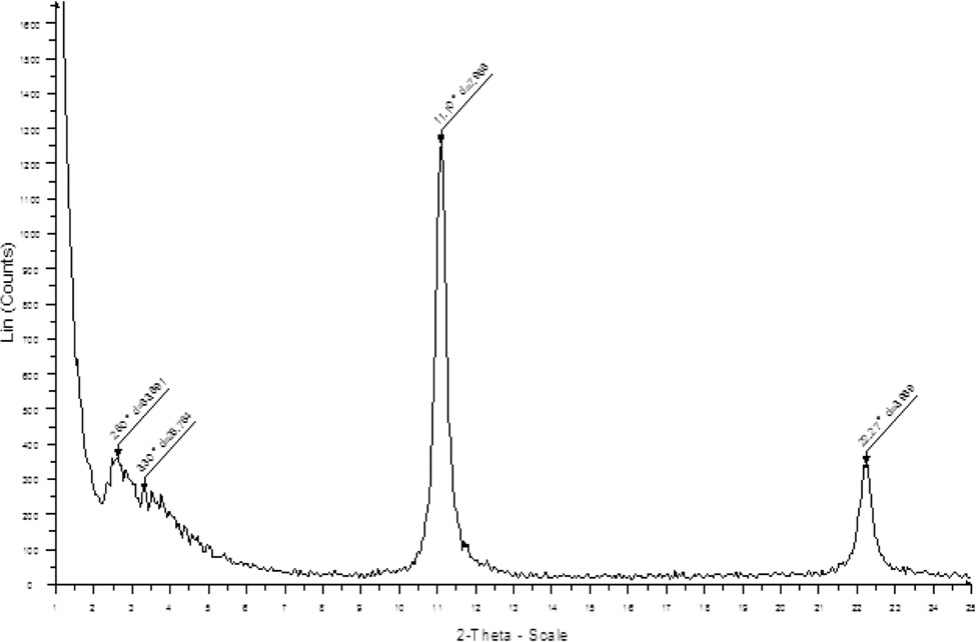
**Powder x-ray diffraction pattern of pvu4a DNA absorbed on 300 meq/100 g HT grown at 150°C.**

With the high absorption observed in these experiments, the manner in which the DNA was absorbed was important to confirm. In order to accomplish this a set of equilibrations at very high DNA concentrations were conducted. In these experiments 2 mg Pvu4a DNA was equilibrated with 2 mg 300 meg/100 g HT that had been grown at 150°C. The mixture was equilibrated while shaking 15 minutes followed by centrifugation at 21,000 rpm. The pellet at the bottom of the centrifuge tube was then scraped from the tube and smeared on a zero background silicon wafer and air dried. The sample was then x-rayed to obtain a powder diffraction pattern given in Figure  7.

It can be seen that the diffraction pattern contains two types of peaks. The first is the typical peak that is from the unintercalated HT at about 0.79 nm. The second set of peaks at approximately 2.5 and 3.4 nm is strong evidence for DNA intercalation. The peak at 2.5 nm represents a 2 nm diameter DNA molecule and 0.5 thick HT layer. The peak at 3.4 nm may be a hydrated complex or a more complex second order peak from a multilayer intercalate. It is interesting to note that both unintercalated and intercalated peaks are present in the diffraction pattern. This can be interpreted in two ways. The first would be that when DNA begins to intercalate the gallery is essentially unzipped and the gallery is completely filled. Alternatively, the DNA only intercalates around the edges leaving the central part of the plates intact. Unfortunately the x-ray data cannot differentiate between these two cases.

## Conclusions

In the current study, the ability of various HTs to bind single- and double-stranded DNA oligonucleotides and circular plasmid DNA was analyzed using absorbance spectroscopy. The binding of DNA to HT for all experiments resulted in almost complete adsorption at 10 mg/ml HT with the exception of plasmid DNA bound to HT synthesized at 25°C, which had 18.5% less DNA bound than HT synthesized at 80°C and 35.2% less than HT at 150°C. This complete adsorption makes it difficult to discern any trends. However, when HT was used at 2 mg/ml, an increase in temperature of HT synthesis resulted in a significant increase of DNA binding for all three nucleic acids. This was expected because the size of HT platelets increases as temperature of HT synthesis increases, giving the plates a larger surface area to bind DNA [23]. The sorption of carbonate by the RT material may also play a role in lower adsorption of DNA. The plasmid DNA exhibited a smaller increase in binding between all three HT samples, compared to both oligomers, due likely to the larger size of the plasmid (4,887 bp vs. 25 bp). Lower concentrations of HT (0.40 and 0.08 mg/ml) resulted in very low percent binding of all three nucleic acids but still showed a slight increase as temperature was elevated. Hydrotalcites used in biomolecule and drug binding studies are frequently synthesized at RT. Our findings strongly suggest that this synthesis temperature is not optimum, especially for biomolecules, and that there is great room for improvement in the use of HT as a delivery vehicle.

HT synthesized with various AECs demonstrated that the maximum DNA binding occurred at 300 meq/100 g, with an HT concentration of 0.4 mg/ml showing the most pronounced effect. No additional DNA was bound when 400 and 500 meq/100 g HT was tested. In fact there was a decrease above 300. At first glance it seems peculiar that the higher charge density HT did not increase adsorption. If the DNA was simply crowding the surface at high loadings, one would expect that there would be a plateau effect in sorption. From a geometric point of view it is instructive to determine how much of the surface of HT is covered for the lowest dose of HT. In the case of the 0.08 mg/ml of HT the amount of surface area per experiment is 0.042 m^2^ based upon a mass of 24 micrograms of HT and a surface area of 1750 m^2^/g. For the dsDNA the geometric dimensions should be a cylinder that is 8 nm long with a diameter of 2 nm. If we project this cylinder on to a surface it would occupy 16 nm^2^. Assuming a molecular weight of 16,250 for the 25 mer (25 bp × 650 g/mole per bp) and that the solution contains 15 micrograms of dsDNA, then if all the dsDNA were to sorb onto the surface it would occupy 0.009 m^2^. This is only 21% of the surface available. The maximum amount absorbed in any experiment at this HT dose level was roughly 20%, so in this case only about 4% of the available surface area is covered. For all other doses, the area covered will be much lower and therefore geometric crowding on the surface is not a factor.

The above calculations suggest that matching the charge density of the HT and DNA could be the controlling factor. This is not a trivial calculation to do since the hydrotalcite plates could be modeled as a flat sheet and the DNA as a cylinder. The cylinder and sheet will only interact partially, being constrained by geometry, but one can calculate the charge density on the basis of charge per square nanometer. Applying this calculation to dsDNA and treating it as a cylinder that is 8 nm long with a diameter of 2 nm yields a surface area of the cylinder of 50.24 nm^2^. With 50 phosphate groups in a dsDNA 25mer, dividing by the surface area indicates that the charge density is 0.99 negative charge per nm^2^. For HT, using the level of aluminum substitution from Table 1 to calculate the charge per unit cell and using the dimensions of the brucite unit cell to calculate the surface area of one face of a unit cell, the charge densities for 100, 200, 300, 400, and 500 meq/100 g are 0.7, 1.4, 2.1, 2.7, and 3.45 cationic charges per nm^2^_,_ respectively.

From the experimental data the optimum charge density for sorption of DNA is 300 meq/100 g. The charge at that point is approximately twice the charge density of the DNA (2.1 vs. 0.99). This makes sense because if the HT plate is pictured as being almost a 2-D sheet, then the charge sites will be compensated for by chloride ions randomly on both sides so that an approaching DNA molecule can displace all of the anions on one side of the plate and not have to compete with any on the opposite side of the plate. This would also allow a DNA on the opposite side of the plate to also have its charge satisfied. At charge densities lower than this the DNA can’t find enough charge sites to satisfy its charge (at 100 meq/100 g) and therefore some of the counter cations will get trapped between the plate and DNA. This would create an excluded area of no charge where DNA molecules would encounter no charges.

As the charge increases to 200 meq/100 g the number of charge sites that have compensating anions on the same side as DNA are insufficient and so for the DNA to be fully charge-compensated then it must compete through the plate for that charge site with chloride on the other side. This will weaken the interaction and create a depleted zone as in the very low charge case. In both of these cases the DNA interaction is weakened. When one goes above the optimum number of twice the charge density the interaction is weakened by excess chloride ions being trapped under the DNA and causing repulsion.

**Figure 8:**
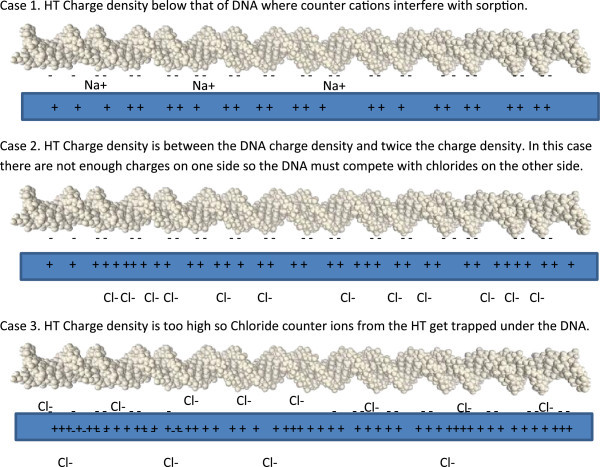
Schematic representation of different adsorption modes of DNA on HT as a function of charge density.

Three of these cases are illustrated in Figure  8. The first case illustrated is when the charge density is well below the charge density of the DNA. Some of the counter cations necessary to satisfy the charge on the DNA will get trapped between the HT and DNA. In the second case, where the charge density of the clay is less than twice the charge density of the DNA, then the DNA must compete with chloride ions on the opposite side of the layer. This competition weakens the bonding of the DNA to the surface. The last case is the situation where the charge density is higher than that of the DNA. In this case excess anions get caught between the layer and the DNA. The optimum case where the charge on the HT is twice that of the DNA is not illustrated but in this case there are no trapped anions or cations and no competition for charge sites with anions on the opposite side of the plate.

The exact method of interaction between DNA-HT (intercalation, exfoliation, or adsorbing to the outer surfaces of the plates) is in dispute. A previous report indicated that plasmid DNA, which is much larger than the previously studied oligomers, adsorbs around the outer surface of HT instead of interacting with the interlayer space [15]. That result is inconsistent with this study since the amount of outer-surface area would be identical for all HT samples at a given HT concentration. This would result in identical DNA binding for each sample rather than increased binding at higher exchange capacities as observed in the current study. In addition, x-ray diffraction data demonstrates that the DNA does indeed intercalate. The only unresolved issue is whether or not the DNA fully unzips the gallery and fills it completely or if the edges are propped open and the interior of the plates are left unintercalated. High resolution transmission electron microscopy might be able to resolve this point.
